# The structure assessment web server: for proteins, complexes and more

**DOI:** 10.1093/nar/gkae270

**Published:** 2024-04-18

**Authors:** Andrew M Waterhouse, Gabriel Studer, Xavier Robin, Stefan Bienert, Gerardo Tauriello, Torsten Schwede

**Affiliations:** Biozentrum, University of Basel, Switzerland; SIB Swiss Institute of Bioinformatics, Computational Structural Biology, Basel, Switzerland; Biozentrum, University of Basel, Switzerland; SIB Swiss Institute of Bioinformatics, Computational Structural Biology, Basel, Switzerland; Biozentrum, University of Basel, Switzerland; SIB Swiss Institute of Bioinformatics, Computational Structural Biology, Basel, Switzerland; Biozentrum, University of Basel, Switzerland; SIB Swiss Institute of Bioinformatics, Computational Structural Biology, Basel, Switzerland; Biozentrum, University of Basel, Switzerland; SIB Swiss Institute of Bioinformatics, Computational Structural Biology, Basel, Switzerland; Biozentrum, University of Basel, Switzerland; SIB Swiss Institute of Bioinformatics, Computational Structural Biology, Basel, Switzerland

## Abstract

The ‘structure assessment’ web server is a one-stop shop for interactive evaluation and benchmarking of structural models of macromolecular complexes including proteins and nucleic acids. A user-friendly web dashboard links sequence with structure information and results from a variety of state-of-the-art tools, which facilitates the visual exploration and evaluation of structure models. The dashboard integrates stereochemistry information, secondary structure information, global and local model quality assessment of the tertiary structure of comparative protein models, as well as prediction of membrane location. In addition, a benchmarking mode is available where a model can be compared to a reference structure, providing easy access to scores that have been used in recent CASP experiments and CAMEO. The structure assessment web server is available at https://swissmodel.expasy.org/assess.

## Graphical Abstract

**Graphical Abstract:**
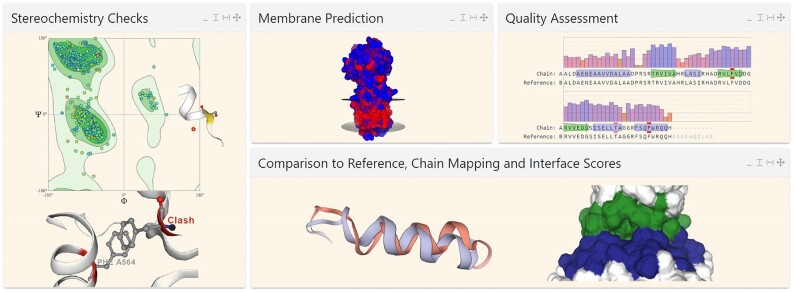

## Introduction

3D structures of macromolecules are essential to understand the mechanisms of cellular processes, including signal transduction, gene expression, and metabolism, thus paving the way for advancements in fields ranging from basic research in structural biology to medicine and biotechnology. Experimental methods such as X-ray crystallography and high-resolution cryo-electron microscopy are employed to generate atomic-level structure representations, which are deposited in the Protein Data Bank (PDB) (1). As experiments are laborious in nature, computational modeling approaches provide an attractive complement. Comparative modeling involves deriving coordinates from structurally similar homologues with known structures. Over the decades, user-friendly pipelines for this process have been developed (2,3). Efforts were not only made for modeling individual targets but also large scale modeling efforts resulting in databases of theoretical models such as the SWISS-MODEL Repository (4,5) and ModBase (6). More recently, deep-learning-based methods such as AlphaFold2 (7), ESMFold (8) and RoseTTAFold (9) were increasingly successful in modeling accurate protein structure models, even in the absence of a clear homologue as a template, which led to the creation of the AlphaFold Protein Structure Database (10) and the ESM Metagenomic Atlas (8).

After CASP14 (11) and the release of AlphaFold2, attention more and more shifted towards challenges beyond tertiary structure prediction. New prediction categories were added to CASP15 (12), with an increased focus on protein complexes (13–15), the introduction of assessment of RNA structure predictions (16) and protein–ligand complexes (17). Assessing macromolecular complexes comes with a set of new challenges ranging from the need for reliable, accurate and fast chain mapping (14) to the development of scores to comprehensively measure the accuracy of these new types of predictions.

In this abundance of structural data, careful assessment of data quality is critical. The wwPDB consortium initiated such efforts by convening task forces specific to the underlying experimental methods to define and recommend relevant quality criteria (18–21). In the case of theoretical models, the provided data on structural quality is even more heterogeneous and dependent on the methodologies used to make the prediction.

In this article, we present a web server dedicated to assessing the quality of structures by providing access to a diverse set of structure evaluation tools in a user-friendly manner. The web server readily accepts any structural model, including those from sources described above.

A user-friendly and fully interactive web view connects sequence with structure information and with results from the structure evaluation tools and allows users to inspect quality indicators of a structure model in an integrated manner. The view includes stereochemistry information (from MolProbity (22) and Ramachandran plots (23)), secondary structure information (from DSSP (24)), global and local model quality assessment of the tertiary structure of protein models with QMEANDisCo (25), and prediction of membrane location. In addition, a benchmarking mode is available where a model can be compared to a reference structure with a comprehensive set of scores that have been used in recent CASP experiments (14–17,26) and CAMEO (27). The scores include assessments of local and global accuracy at the fold level and at interfaces of any type of macromolecular complexes. Hovering and clicking on evaluation results is directly highlighted in the sequence and structure views, which greatly facilitates the visual exploration and evaluation of structure models. Multiple selections can be performed by holding the Control or Command key.

The structure assessment web server is integrated within SWISS-MODEL and has received regular updates since its initial release 5 years ago. In 2024, on average the tool assessed >600 structures every day, emphasizing its usefulness.

## Implementation

### Overview of the web server functionality

**Figure 1.**
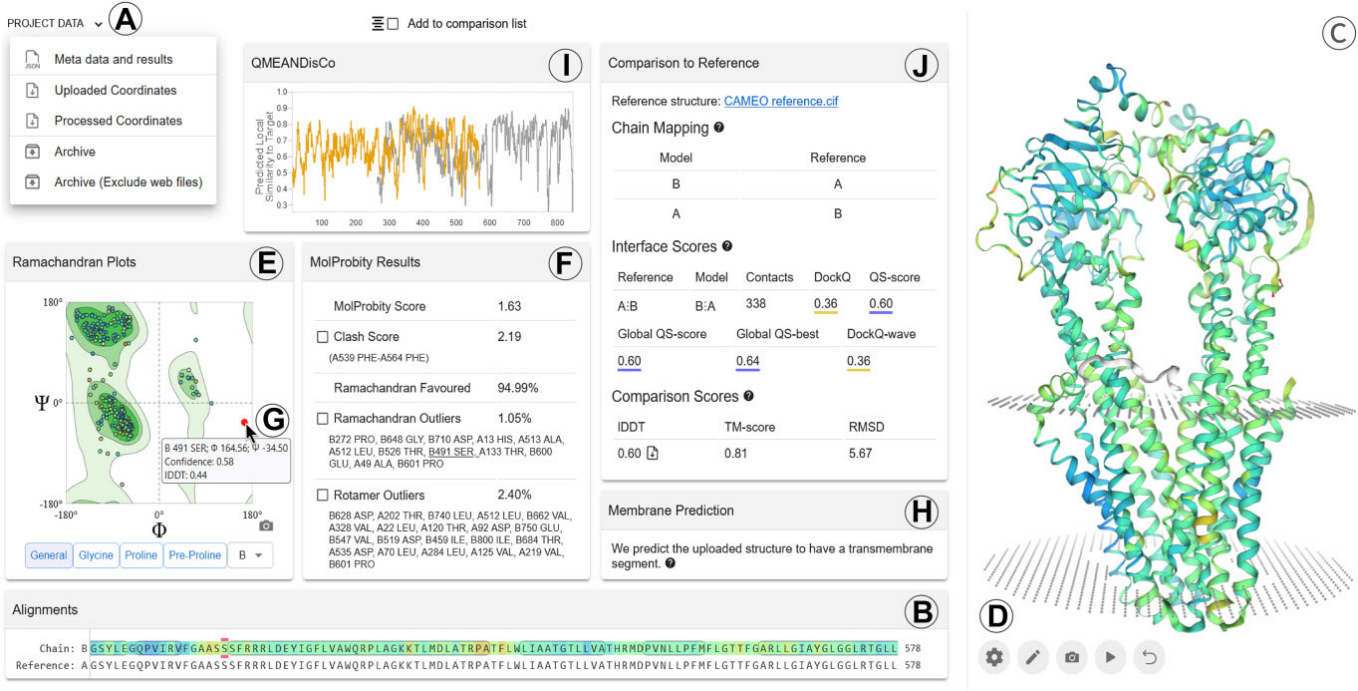
The fully interactive structure assessment workspace. Results can be saved for future reference (**A**). The ‘Alignments’ component (**B**) and 3D viewer (**C**) display the uploaded structure. The structure viewer (**D**) buttons enable customizing the display. Stereochemistry assessment results are available through the ‘Ramachandran Plots’ (**E**) and ‘Molprobity Results’ (**F**) components. Hovering over individual results provides additional information (**G**) and immediately highlights the respective locations in the ‘Alignments’ component (B) as well as the interactive 3D view (C). ‘Membrane Prediction’ (**H**) suggests that the uploaded structure possesses the characteristics of a transmembrane protein which is also displayed in the 3D view. This workspace additionally enabled the ‘QMEANDisCo’ (**I**) component which displays predicted per-residue model qualities, and the ‘Comparison to Reference’ (**J**) component which automatically establishes the most likely chain-to-chain assignment with a user defined reference structure and displays metrics specific for per-residue and full structure similarities as well as oligomeric interface similarities.

The structure assessment web server is a dashboard organized into ‘components’, which display analysis results, and are connected to each other and to a 3D viewer (Figure 1). The dashboard adapts automatically to any given screen resolution and additionally allows the user to rearrange their own display of results to facilitate the analysis. For instance, arranging the alignment of model to reference chain next to the MolProbity results and 3D viewer, facilitates the analysis of outliers in sequence and structure space. In order to keep the interface clean and readable, additional information is revealed in tooltips that appear upon hovering the results.

The structure assessment allows directly uploading any coordinates of a macromolecule or macromolecular complex in legacy PDB format or in the preferred PDBx/mmCIF format (28). This mode offers the most flexibility, with the choice of assessment tools to run and the possibility to upload a reference structure. Uploaded models are validated on the fly upon upload and undergo basic pre-processing to ensure they can be handled properly by the different tools that are offered by the server. Atoms or residues may be removed if they do not follow the nomenclature from the Chemical Component Dictionary from the PDB (29). In addition, biological assemblies are extracted from uploads in the PDBx/mmCIF format, which might result in the renaming of chains. Both the uploaded and pre-processed structure files can be downloaded for further manual inspection.

In addition, assessments can be performed directly from the SWISS-MODEL interactive modeling workbench. SWISS-MODEL provides QMEANDisCo as quality assessment for homology models (25,30), and predicted lDDT (pLDDT) (7) for models based on templates from the AlphaFold Database (10). However, around 20% of users forward their models to the structure assessment server in order to gain additional annotation from MolProbity, the Ramachandran plot and simplified display of target-template alignment. Finally, users can also run the structure assessment directly from the SWISS-MODEL Repository (SMR). This is particularly useful to assess experimental structures from the PDB or models from AlphaFold Database and ModelArchive (https://modelarchive.org/) as SMR displays structures from these resources per UniProtKB identifier. Currently, around 90% of assessments are started directly from SWISS-MODEL and SMR.

Once the analysis is completed, the results can be archived either as a fully interactive package to be uncompressed and displayed in a web browser directly from the local file system, or as a simpler package containing the results in JSON format together with the input coordinates file(s). Both are accessible from the ‘Project Data’ button (Figure 1A). Results are kept on our servers for a duration of 2 weeks. They can be accessed and shared by keeping track of their URL. Optionally, users can supply an email address or register an account to easily keep track of their results across browsers and computers. This website is free and open to all users and there is no login requirement.

### Sequence and 3D viewers

The structure assessment dashboard provides a direct view of the sequence (‘Alignments’ component, Figure 1B) and three dimensional structure (Figure 1C) of the submitted model. The default 3D viewer is NGL (31). The two components are tightly integrated: hovering a residue in the structure highlights it in the sequence viewer, while clicking on the sequence centers onto the residue. It is also possible to select a stretch of sequence to zoom the 3D viewer to it. This allows to quickly identify the relationship between the sequence and the structure. Coloring can be controlled from the sequence and 3D viewer settings button (‘cogwheel’ button, Figure 1D) and affects both views simultaneously. Several color schemes are available, from quality estimates (model confidence or B-factors) to chemical properties or secondary structure.

The contents of the ‘Alignments’ component varies depending on the source of the model. In case a reference structure was uploaded alongside the model, the ‘Alignments’ component displays the reference-model alignment. Extra controls in the 3D viewer settings (Figure 1D) allow tuning the opacity of the reference structure to ease the analysis. If the assessment was started from the SWISS-MODEL workbench, the target-template alignment is displayed. In all other cases, the sequence extracted from the structure file is displayed in place of an actual alignment.

Finally, structures can be interactively annotated which enable custom coloring of specific residues and motifs, adding custom labels or highlighting side-chains of specific residues.

### Stereochemistry analysis

Two web server components facilitate the evaluation of physical viability: ‘Ramachandran Plots’ (Figure 1E) and ‘MolProbity Results’ (Figure 1F).

A Ramachandran plot (23) provides an assessment of the overall plausibility of protein conformation, achieved by comparing the backbone dihedral angles of a structure with data extracted from 12 521 non redundant experimental structures (pairwise sequence identity cutoff 30%, X-ray resolution cutoff 2.5Å) as culled from PISCES (32). The contour plots allow rapid identification of unlikely conformations similar to other tools such as PROCHECK (33) or MolProbity (22) but seamlessly integrate with other web server components. The plot is directly linked to the ‘Alignments’ component (Figure 1B) and the 3D viewer (Figure 1C), so that hovering a point highlights the residue in these components, enabling the quick localisation of outliers in the sequence and structures. In addition, a tooltip displays additional information and results about the residue (Figure 1G).

Conversely, MolProbity (22) enables a more fine-grained all-atom stereochemistry validation for both proteins and nucleic acids. The structure assessment web server runs MolProbity version 4.4 as available from https://github.com/rlabduke/MolProbity and displays a selection of the most important validation results, including an overall score which reflects the crystallographic resolution at which such a quality would be expected. Additional details about the meaning of each of the scores and their interpretation is available from the structure assessment help page. Just like the Ramachandran plot, hovering an item in the displayed results (Figure 1F) directly highlights the problematic residue(s) in the sequence and 3D viewers and displays a tooltip with detailed information.

### Membrane prediction

The ‘Membrane prediction’ component (Figure 1H) computes the most likely membrane location based on structural information using the membrane finding algorithm of the QMEANBrane tool (34) which is based on the solvation model described for the Orientations of Proteins in Membranes database (35). The results serve as input for a classification based on energetic and geometric criteria which aims at minimizing false positives. A full benchmark and evaluation is available in Supplementary Data 1. If a transmembrane segment is likely, it gets displayed in the 3D viewer. The full parameters are available in the downloadable JSON file.

### Quality estimates

Quality estimates of theoretical models are an indispensable ingredient of any modeling pipeline as the expected accuracy of a theoretical model ultimately determines its usefulness for the biological question at hand. Structure models often come with their own quality estimates. For instance, models from AlphaFold2 or ESMFold contain a per-residue pLDDT estimate which can be visualized by selecting one of the ‘Confidence’ color schemes (Figure 1D). Homology models from SWISS-MODEL or SMR display a similar per-residue confidence score, QMEANDisCo.

In addition, the ‘QMEANDisCo’ component (Figure 1I) can be enabled for any homology-based protein model by activating the corresponding checkbox upon upload. It provides quality estimates for global model quality as well as per-residue estimates using the QMEANDisCo method (25). Per-residue estimates are visualized in the ‘Alignment’ component with a color gradient which can also be mapped as ‘Confidence’ color scheme onto the model in the 3D viewer to identify problematic regions. QMEANDisCo strongly relies on template information and should be analyzed with care for *de novo* methods such as AlphaFold2.

AlphaFold2 has introduced a new type of quality estimate, the Predicted Aligned Error (PAE), which no longer estimates the accuracy of single residues, but measures the confidence in the relative position of two residues within the predicted structure. While the PAE provides valuable information about the prediction, we decided not to include it in our assessment as it is very specific to AlphaFold2 models and has been well handled elsewhere (36).

### Comparison to reference

By selecting the ‘Compare to reference’ checkbox, users can activate the respective component (Figure 1J) to compare theoretical models of macromolecular complexes to experimentally determined references for benchmarking purposes. This enables access to a comprehensive set of scores that have been used in recent CASP experiments (14–17,26) and CAMEO (27). They focus on local per-residue similarity (lDDT), global structure similarity (lDDT, RMSD, TM-score), and interface-specific similarity (QS-score, DockQ). While most of the scores have been designed with proteins in mind, most are useful also for the analysis of RNA structures. The component is split into three parts for which we provide a short summary in this manuscript. More detailed information can be found in the structure assessment server help page (https://swissmodel.expasy.org/assess/help).

**Chain mapping**: A one-to-one correspondence between model and reference chains is a prerequisite for scoring. The underlying algorithm was used in CASP15 (14,15) and aims to provide the optimal mapping with respect to the superposition independent QS-score (37). The mapped chains are displayed in a table which is connected with the 3D viewer for visualization and interactive exploration of the mapping. Non-mapped chains, for example in case of different stoichiometries in model and reference, are displayed separately.

**Comparison scores** assess the similarity between the model and reference structures. The displayed lDDT, TM-score and RMSD report on different aspects of structural similarity.

The local distance difference test (lDDT) (38) is a full atomic score and assesses the differences in interatomic distances between model and reference structure. It is thus inherently superposition free. As opposed to the original publication, lDDT displayed here natively supports oligomers as well as RNA/DNA for which it was extensively tested (15,16,39). Interatomic distances across interfaces get treated as any other pairwise distance. The global lDDT for the full model is displayed in the result table. Per-residue lDDT values are available in the interactive sequence display, which also allows to map lDDT as a color gradient onto the model in the 3D viewer by clicking on the ‘Display options’ (‘cogwheel’) icon in the display settings at the bottom left corner of the viewer (Figure 1D). If stereochemical irregularities are present that affect lDDT, they are displayed right underneath the global lDDT score and can be explored interactively.

TM-score (40) as computed with USalign (41) assesses the topological similarity of macromolecular complexes including RNA. It relies on a global superposition and considers backbone coordinates (Cα for amino acids, C3’ for nucleotides).

RMSD uses backbone coordinates (Cα, C3’) from all mapped residues in the full complex to derive a Kabsch superposition (42) which inherently comes with the respective RMSD value.

**Interface scores** complement measures of general structural similarity with interface centric similarity measures. The displayed global QS-score, the QS-best variant and DockQ-wave are color coded to provide a visual guide for model similarity. Additionally, a table which is linked with the 3D view allows users to interactively explore each protein-protein interface with their respective interacting chains, numbers of residue contacts, and per-interface QS- and DockQ scores.

QS-score (37) quantifies the similarity between interfaces as a function of interface contacts. QS-score can be computed for individual interfaces but the main intention is to aggregate contributions from all interfaces to get a single score of the full complex. QS-score has been updated to incorporate contacts involving nucleotides which enables chain mapping also with RNA and DNA. QS-best only considers the part of the structure that is present in both the model and the reference. This can be useful if the model is a substructure of the reference, or if the reference is not fully resolved.

DockQ (43) combines the established protein-protein interface similarity metrics *F*_nat_ (conserved fraction of native contacts), iRMS (interface RMSD) and LRMS (ligand RMSD) from the Critical Assessment of PRedicted Interactions (CAPRI) community (44) into one continuous score. It strictly processes single protein–protein interfaces but is complemented by DockQ-wave (14) which scores full protein complexes as a weighted average of per-interface DockQ scores. Protein-nucleotide interfaces do not affect DockQ-wave and weights are the number of native inter-chain contacts of each interface in the reference structure.

All described metrics are computed with the ‘compare-structures’ action (https://openstructure.org/docs/actions/) of the OpenStructure computational structure biology framework (45). The action provides a full benchmarking suite and can be executed locally for large scale analysis.

### Similar tools

Several web servers and desktop tools provide a similar or overlapping set of features, here we list some of the most relevant ones. MolProbity (22) is the gold standard tool for stereochemistry analysis. A web server is provided to avoid the need to install the software. While the MolProbity web server provides additional capabilities (such as basic structure manipulation tools like adding hydrogens, filling small loops and NQH flips) and reports more detailed information, it does not provide an interactive, direct integration of stereochemical check results to 3D and sequence viewers. The ProteinPlus web server (46) provides a collection of related structure analysis and modeling tools, with a focus on protein-ligand complexes. PDBsum (47) is a database of detailed structure analysis of entries from the PDB and the AlphaFold Database and allows upload of arbitrary models. Analysis includes non-interactive sequence analysis as well as a Ramachandran plot. Models from the AlphaFold Database are colored according to the attached pLDDT. In addition, several molecular graphics desktop applications such as Coot (48) and UCSF ChimeraX (49), as well as the Phenix package (50), enable somewhat similar analyses with Ramachandran plots and stereochemistry checks, together with a direct interactivity with the 3D viewer. It should be noted that the comparison to a reference is a unique feature of the structure assessment web server and the underlying OpenStructure tool.

## Conclusions

The structure assessment web server is a one-stop shop that tightly integrates an array of state-of-the-art tools that facilitate the analysis of the quality and accuracy of structural models. Stereochemistry analysis with Ramachandran plots and MolProbity analysis report on the physical viability of the model. Quality estimates are either displayed or computed for homology models and inform on the estimated accuracy of the predictions. By uploading a reference structure, a benchmarking mode scores the model coordinates against a known structure. Sequence and 3D structure viewers, together with the prediction of membrane location, connect all these results in an interactive and user-friendly interface that allows the visual exploration and evaluation of structure models.

The structure assessment web server is highly extensible. We are working on new components to be added such as display and scoring of protein–ligand interactions as well as additional sequence annotation.

## Supplementary Material

gkae270_Supplemental_File

## Data Availability

The Structure Assessment web server is available at https://swissmodel.expasy.org/assess. This website is free and open to all users and there is no login requirement.
