# Supplementary material for: The structure assessment web server: for proteins, complexes and more
Source: Nucleic Acids Res. 2024 Apr 18;52(W1):W318–23. doi: 10.1093/nar/gkae270 (PMC11223858; doi:10.1093/nar/gkae270)
Supplement: gkae270_Supplemental_File [file gkae270_supplemental_file.pdf]

The Structure Assessment Web Server: for Proteins, Complexes and More

Supplementary Data

Andrew M. Waterhouse[1,2], Gabriel Studer[1,2], Xavier Robin[1,2], Stefan Bienert[1,2], Gerardo Tauriello[1,2], Torsten Schwede[1,2]

Affiliations:

1. Biozentrum, University of Basel, Switzerland
2. SIB Swiss Institute of Bioinformatics, Computational Structural Biology, Basel, Switzerland

Automated identification of transmembrane protein structures

PDB IDs of transmembrane proteins were identified by querying the Orientations of Proteins in Membranes database (1) in September 2020. Structures with at least one secondary structure element spanning the full membrane plane were considered positives, thus filtering monotopic and peripheral membrane proteins. During the evaluation process, 258 additional PDB entries that clearly represent transmembrane proteins were manually added to this list upon manual inspection, leading to a total of 5713 PDB ids representing transmembrane proteins.

Evaluation has been performed on all biological assemblies of the SWISS-MODEL template library (2) in September 2020. Incomplete biological assemblies of viral capsids often have large hydrophobic patches and are prone to misclassification. Biological assemblies of viral capsids have thus been dropped, leading to a total of 242530 biological assemblies with 7081 labeled as positives, i.e. transmembrane and 235449 as negatives.

Features for classification are derived from the membrane finding algorithm described for the QMEANBrane tool (3) which is based on the solvation model described for the Orientations of Proteins in Membranes database (4). The code is freely available as part of the OpenStructure computational structural biology framework (5). The following features were computed:

- e: Energy term derived from membrane finding algorithm
- asa: Total membrane accessible surface in Å^2
- w: The computed width in Å of the membrane
- n_sheet, n_helix: Number of extended or helical secondary structure elements that span the computed membrane

- n_sheet_aligned, n_helix_aligned: Fraction of these secondary structure elements that exhibit an angle < 50° degrees with the computed membrane normal. The direction vector of a secondary structure element is determined as the direction of largest variance with respect to Cα carbon atoms and is computed with a principal component analysis.

Classification parameters were manually chosen in order to minimize the false positive rate. A biological assembly is classified as positive if all the following criteria are met:

- e < -30’000.0
- e/asa < -8.0
- w > 18.0

In addition, depending on the type of transmembrane structure, one of the following statements must also hold true:

- n_sheet_aligned > 0.5 if n_sheet > n_helix
- n_helix_aligned > 0.5 otherwise

This gives the following classification performance on the evaluated assemblies:

True positives:	6643
False positives:	6
True negatives:	235443
False negatives:	438
**Precision:**	**0.999**
**Recall:**	**0.938**

References

1. Lomize,M.A., Pogozheva,I.D., Joo,H., Mosberg,H.I. and Lomize,A.L. (2012) OPM database and PPM web server: resources for positioning of proteins in membranes. *Nucleic Acids Res.*, **40**, D370–6.

2. Waterhouse,A., Bertoni,M., Bienert,S., Studer,G., Tauriello,G., Gumienny,R., Heer,F.T., de Beer,T.A.P., Rempfer,C., Bordoli,L., *et al.* (2018) SWISS-MODEL: homology modelling of protein structures and complexes. *Nucleic Acids Res.*, **46**, W296–W303.

3. Studer,G., Biasini,M. and Schwede,T. (2014) Assessing the local structural quality of transmembrane protein models using statistical potentials (QMEANBrane). *Bioinformatics*, **30**, i505–11.

4. Lomize,A.L., Pogozheva,I.D., Lomize,M.A. and Mosberg,H.I. (2006) Positioning of proteins in membranes: a computational approach. *Protein Sci.*, **15**, 1318–1333.

5. Biasini,M., Schmidt,T., Bienert,S., Mariani,V., Studer,G., Haas,J., Johner,N., Schenk,A.D., Philippsen,A. and Schwede,T. (2013) OpenStructure: an integrated software framework for computational structural biology. *Acta Crystallogr. D Biol. Crystallogr.*, **69**, 701–709.
